# Localization Microscopy (SPDM) Reveals Clustered Formations of P-Glycoprotein in a Human Blood-Brain Barrier Model

**DOI:** 10.1371/journal.pone.0044776

**Published:** 2012-09-12

**Authors:** Olga Huber, Alexander Brunner, Patrick Maier, Rainer Kaufmann, Pierre-Olivier Couraud, Christoph Cremer, Gert Fricker

**Affiliations:** 1 Institute of Pharmacy and Molecular Biotechnology, University of Heidelberg, Heidelberg, Germany; 2 Kirchhoff-Institute for Physics, University of Heidelberg, Heidelberg, Germany; 3 Department of Medical Physics in Radiology, German Cancer Research Center (DKFZ), Heidelberg, Germany; 4 Department of Radiation Oncology, Mannheim Medical Centre, University of Heidelberg, Mannheim, Germany; 5 Department of Biochemistry, University of Oxford, Oxford, United Kingdom; 6 Department of Cell Biology, Université Paris Descartes, Paris, France; 7 Institute of Molecular Biology, Mainz, Germany; Biological Research Centre of the Hungarian Academy of Sciences, Hungary

## Abstract

P-glycoprotein (Pgp; also known as MDR1, ABCB1) is the most important and best studied efflux transporter at the blood-brain barrier (BBB); however, the organization of Pgp is unknown. The aim of this study was to employ the recently developed super-resolution fluorescence microscopy method spectral precision distance microscopy/spectral position determination microscopy (SPDM) to investigate the spatial distribution of Pgp in the luminal plasma membrane of brain capillary endothelial cells. Potential disturbing effects of cell membrane curvatures on the distribution analysis are addressed with computer simulations. Immortalized human cerebral microvascular endothelial cells (hCMEC/D3) served as a model of human BBB. hCMEC/D3 cells were transduced with a Pgp-green fluorescent protein (GFP) fusion protein incorporated in a lentivirus-derived vector. The expression and localization of the Pgp-GFP fusion protein was visualized by SPDM. The limited resolution of SPDM in the z-direction leads to a projection during the imaging process affecting the appeared spatial distribution of fluorescence molecules in the super-resolution images. Therefore, simulations of molecule distributions on differently curved cell membranes were performed and their projected spatial distribution was investigated. Function of the fusion protein was confirmed by FACS analysis after incubation of cells with the fluorescent probe eFluxx-ID Gold in absence and presence of verapamil. More than 112,000 single Pgp-GFP molecules (corresponding to approximately 5,600 Pgp-GFP molecules per cell) were detected by SPDM with an averaged spatial resolution of approximately 40 nm in hCMEC/D3 cells. We found that Pgp-GFP is distributed in clustered formations in hCMEC/D3 cells while the influence of present random cell membrane curvatures can be excluded based on the simulation results. Individual formations are distributed randomly over the cell membrane.

## Introduction

The primary obstacle to the central nervous system (CNS) is the blood-brain barrier (BBB), which is formed by the brain capillary endothelial cells. These cells express multiple membrane-bound ATP-binding cassette (ABC) efflux transporters including P-glycoprotein (Pgp, ABCB1), breast cancer resistance protein (BCRP, ABCG2), and several isoforms of multidrug resistance-associated proteins (MRPs, ABCCs). They prevent the entry of xenobiotics and potentially toxic metabolites into the CNS and contribute to lowered drug accumulation within the brain [1], [2], [3], [4].

**Figure 1:**
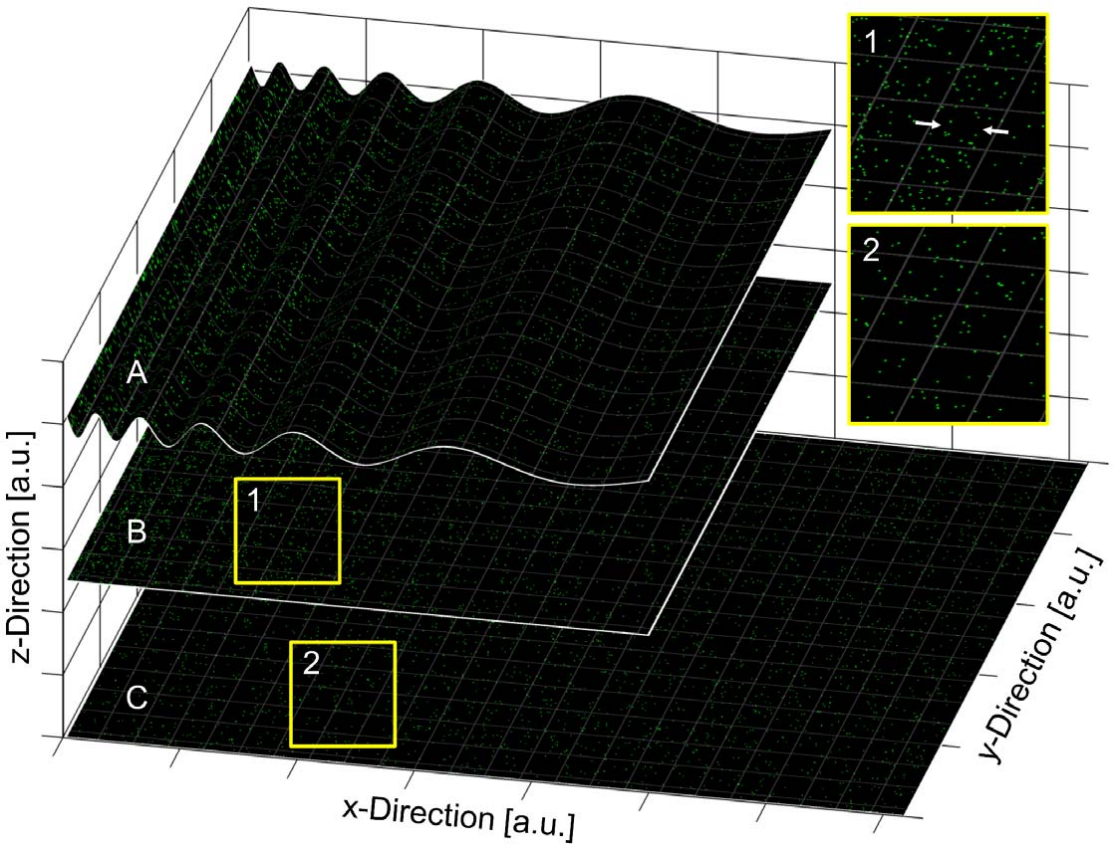
Simulated cell curvature effects on projected random molecule distributions. **A:** Random molecule distribution on a chirp curvature in x-direction. **B:** Projection of the curved random distribution along the z-direction. **C:** Non-curved random molecule distribution (same as in A). Molecule aggregations along the x-direction are induced by the projection of the curved random distribution (highlighted by two arrows in section 1). These aggregations are not present in the non-curved random distribution (section 2). In a real SPDM measurement the projection is caused by a non-improved z-resolution.

P-glycoprotein, which is the most important and best studied efflux transporter at the BBB, is localized within the luminal membrane of brain capillaries [5]. It is an integral membrane protein consisting of two subunits with together 12 transmembrane segments and two nucleotide binding domains. A linker between the N- and C-terminal halves includes phosphorylation sites to regulate the activity of the export pump. The protein recognizes a remarkably broad diversity of molecules ranging from amphiphilic, to neutral or cationic structures [6]. It is still not completely clear whether the transported substrates are released in the exoplasmic leaflet of a membrane or directly into the extracellular medium. There is evidence that Pgp transports its substrates after binding within the inner leaflet of a membrane [7], [8]. As reviewed [9], Pgp is highly sensitive to its lipid environment, and the fluidity of the surrounding lipid rafts directly influences the activity of the export pump.

However, very little is known whether Pgp is organized in a membrane as single molecules, in clustered formations or associated to other proteins. Recently, green fluorescent protein (GFP) labeled Pgp has been used to study intracellular and membrane trafficking of the protein: A Pgp-GFP fusion protein transfected in liver-derived cells was localized both in the canalicular membrane and in the sub-apical and Golgi regions of polarized cells. Moreover, it has been shown, that this fusion protein was directly transferred from the Golgi to the apical membrane [10]. Further on, newly synthesized Pgp, probed as Pgp-GFP fusion protein, was directly transferred from the Golgi to the apical membrane of polarized HepG2 cells in a cholesterol-sensitive manner [11].

In the present study we used a Pgp-GFP fusion protein as a probe to determine the spatial distribution of Pgp within the luminal membrane of brain capillary endothelial cells using immortalized human cerebral microvascular endothelial cells (hCMEC/D3) [12] as a cellular model of the BBB. These cells form confluent monolayers and exhibit the expression of BBB endothelial cell characteristics, for instance factor-VIII-related-antigen or ABC-transporters, e.g. Pgp.

**Figure 2:**
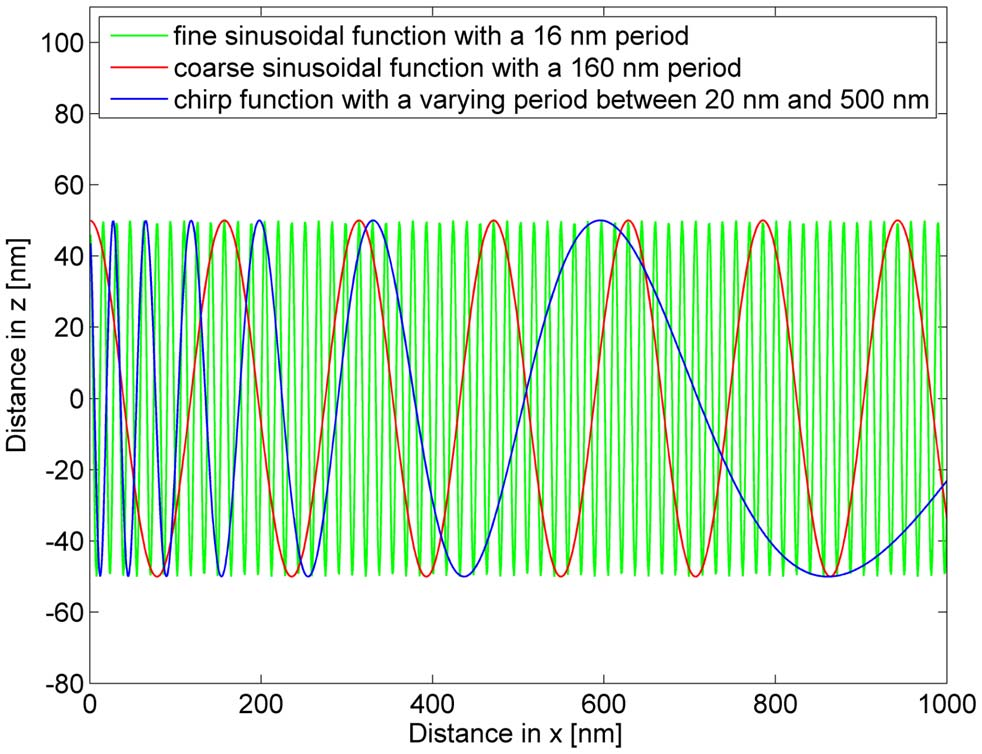
The three different functions to simulate the curvature of a cell. All functions have the same amplitude of 100 nm and are applied only along the x-direction. The fine sinusoidal function has a period of approximately 16 nm and the coarse sinusoidal function of approximately 160 nm. The chirp function has a sin (1/x) form with a varying period.

Biological studies of subcellular structures in living cells or tissue with fluorescence microscopes is advantageous because it is non-invasive and specific. However, conventional microscopes such as wide-field or confocal microscopes are limited in spatial resolution and are not able to resolve structures smaller than 200 nm in the imaging plane. This resolution limit is caused by the diffraction limit of light and is often referred as the Abbe limit. However, a distribution analysis of Pgp-GFP on a single molecular base is possible with localization microscopy, if the localization accuracy and the number of detected molecules is sufficient. In this work, visualization of expression and localization of the Pgp-GFP fusion protein was studied by super-resolution fluorescence microscopy (spectral precision distance microscopy/spectral position determination microscopy; SPDM). SPDM is a technique of far field localization microscopy [13], [14], [15], [16], [17], [18] which allow a structural resolution far below conventional confocal fluorescence microscopy. Generally, localization microscopy is based on the determination of the positions of single molecules/point sources using appropriate fluorescence emission characteristics useful for photonic discrimination of the emitters. In the SPDM mode used here, a light induced long-lived dark state was applied for optical isolation of their signals [17], [19]. The software-supported reconstruction combines the coordinates of single points to a complete image. Thus, a lateral resolution of 20–40 nm becomes possible. By additional algorithms, the single molecule coordinates can be used to determine and analyze small molecule aggregations down to a cluster size of few tens of nm [20]. SPDM is advantageously because standard fluorophores, e.g. GFP, can be used and minimally only one laser wavelength for excitation and switching is needed. It is important to mention that the type of SPDM used here does not improve the resolution in the z-direction. Recently, methods for single molecule detection with a z-resolution below the Abbe limit have been reported [21], [22]. However, this resolution increase is still practically limited to the 50–200 nm range and therefore still not capable to determine very small cell membrane curvatures. The limited resolution results in a projection over the excitation volume along the z-direction during the imaging process. This affects the measured spatial distribution of fluorescence molecules in super-resolution SPDM images. To address this problem, simulations of molecule distributions on differently curved cell membranes were performed and their projected spatial distribution was investigated. Furthermore, highly curved cell boundaries have been excluded from the spatial distribution analysis.

**Figure 3:**
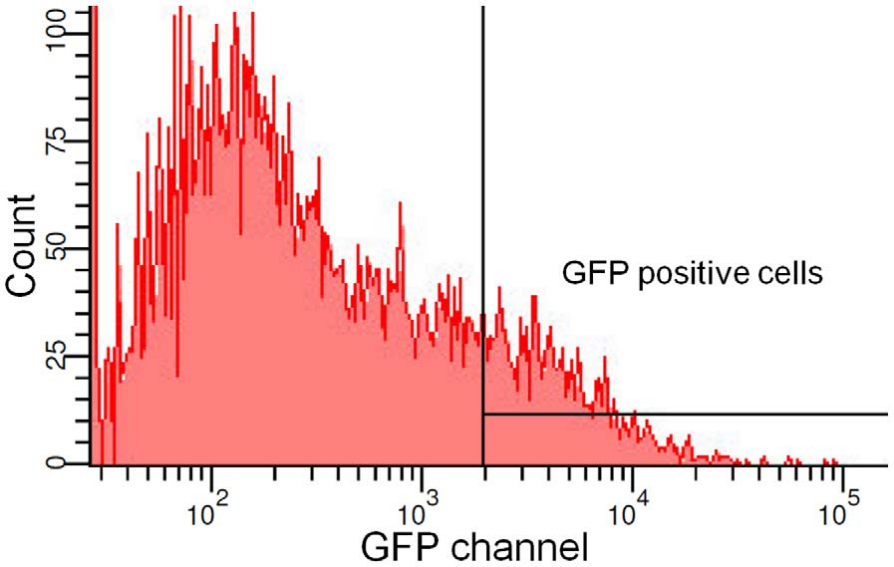
Flow cytometry of transduced hCMEC/D3 cells. Approximately 11% of transduced cells show GFP expression.

## Methods

### 2.1 Cell culture

The immortalized human brain endothelial cell line hCMEC/D3 [12] was grown in EBM-2 medium (Lonza, Basel, Switzerland) supplemented with fetal bovine serum, Penicillin/Streptomycin, ascorbic acid, hydrocortisone, basic FGF, HEPES and Chemical Defined Lipid Concentrate. Cells were seeded on rat tail collagen-coated (Roche, Mannheim, Germany) 75 cm^2^ flasks and 8-well Permanox chamber slides (Thermo Fisher Scientific, Langenselbold, Germany) for microscopic studies, respectively. One day prior flow cytometry experiments, the medium was changed to phenol red-free EBM-2 medium (Promocell, Heidelberg, Germany) to avoid fluorescence disturbance.

All cells were maintained at 37°C in humidified air with 5% CO_2_. Cell medium was changed every 2–3 days.

**Figure 4:**
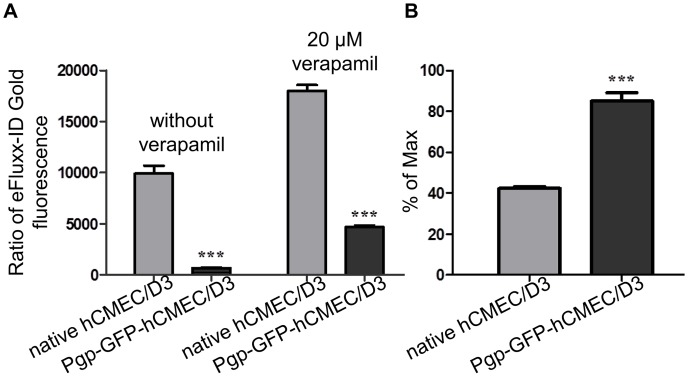
Intracellular accumulation of eFluxx-ID Gold in nontransduced and Pgp-GFP-hCMEC/D3 cells. **A:** Quantitative analysis of the intracellular fluorescence intensity of eFluxx-ID Gold in native and in transduced Pgp-GFP-hCMEC/D3 cells in the absence or presence of 20 µM verapamil. **B:** The multidrug resistance activity of Pgp-GFP-hCMEC/D3 cells is increased by a factor of 2 compared to nontransduced cells, which was determined by calculating the multidrug resistance factor for each probe.

### 2.2 Vector Construction

For cloning of the lentiviral vector pHR’SlN-Pgp-GFP the cDNA of GFP was amplified by PCR using pTagGFP2-N (Evrogen, Moscow, Russia) as template and primers containing specific restriction sites (5′ Xho I–GFP–Xba I 3′). The sequence was confirmed by cycle sequencing (GATC, Konstanz, Germany). Then, the cDNA of GFP was cloned via Xho I/Xba I in frame 3′ of the cDNA of Pgp (lacking the stop codon) already located in pBluescript (Maier, unpublished results). The fusion gene Pgp-GFP was finally inserted via BamH I/Xba I into the lentiviral vector pHR’SIN-IRES-EGFP [23] replacing IRES-EGFP.

### 2.3 Virus Production and Viral Transduction

Lentiviral supernatant was produced and titrated as described previously [24]. The hCMEC/D3 cells were transduced once with a multiplicity of infection (MOI) of 10 at a density of 2×10^5^ cells/cm^2^ in the presence of 8 µg/ml polybrene. Within 4–6 days after transduction, GFP expressing cells were sorted in a FACSAria cell sorter (Becton Dickinson, Heidelberg, Germany).

**Figure 5:**
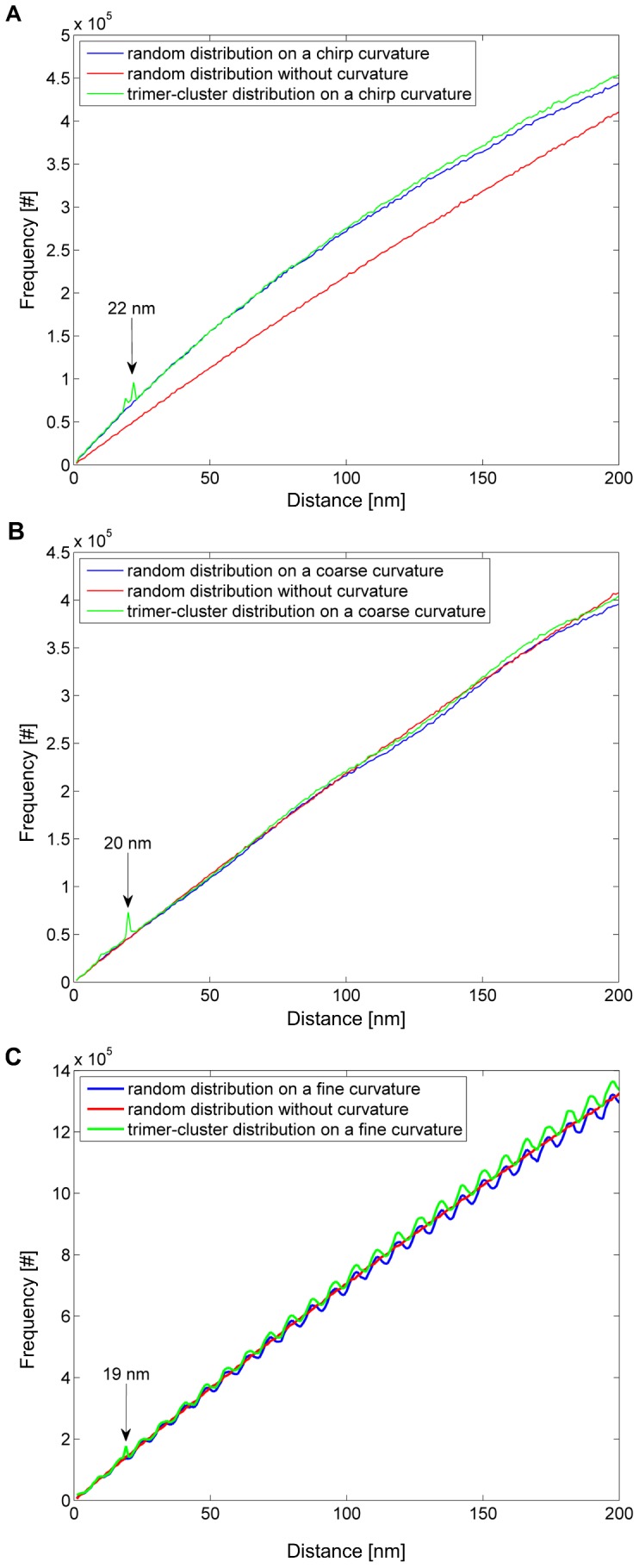
Simulation results of random and cluster molecule distributions on cell curvatures. Spatial distribution analysis of random (blue) and trimer-cluster (green) molecule distributions on **A:** a chirp curvature, **B:** a coarse sinusoidal, and **C:** a fine sinusoidal curvature in x-direction projected along the z-direction (cf. Fig. 2 B). For control, a spatial distribution analysis of a random distribution without a curvature (red) is present in each plot. Only in A and B, the molecule clusters can be detected (highlighted by arrows with peak positions); whereas in C the detection of clusters is no longer possible since the peak at 19 nm cannot be separated from the peak resulting from cell curvature.

### 2.4 Flow Cytometry Analysis and Efflux Assay of Transduced Cells

Flow cytometric assays were performed on a FACSAria (Becton Dickinson, Heidelberg, Germany), mounted with an air-cooled 488 nm argon ion laser. Data was analyzed using FACSDiva software (Becton Dickinson, Heidelberg, Germany). Briefly, cells were trypsinized, resuspended at 1–2×10^6^ cells/ml in phenol red free medium and incubated with the fluorescent probe eFluxx-ID Gold (ENZO Life Sciences, Lörrach, Germany) in a concentration according to manufacturer`s recommendation for 30 min at 37°C, in the absence or presence of 20 µM verapamil and analyzed immediately by flow cytometry. eFluxx-ID Gold is a recently presented xanthene-based small molecule dye developed for monitoring of multidrug resistance (MDR) activity in live cells [25]. eFluxx-ID Gold (λ_ex/em_ = 530/555 nm) fluorescence intensity was measured in the FL2/PE (585/42 nm filter) channel. Gating of viable cells was based on light scatter parameters. Analysis of 1×10^4^ or 3×10^4^ cells per sample was carried out in the fluorescence/count four decades log diagram, collecting the autofluorescence signal in the first decade.

**Figure 6:**
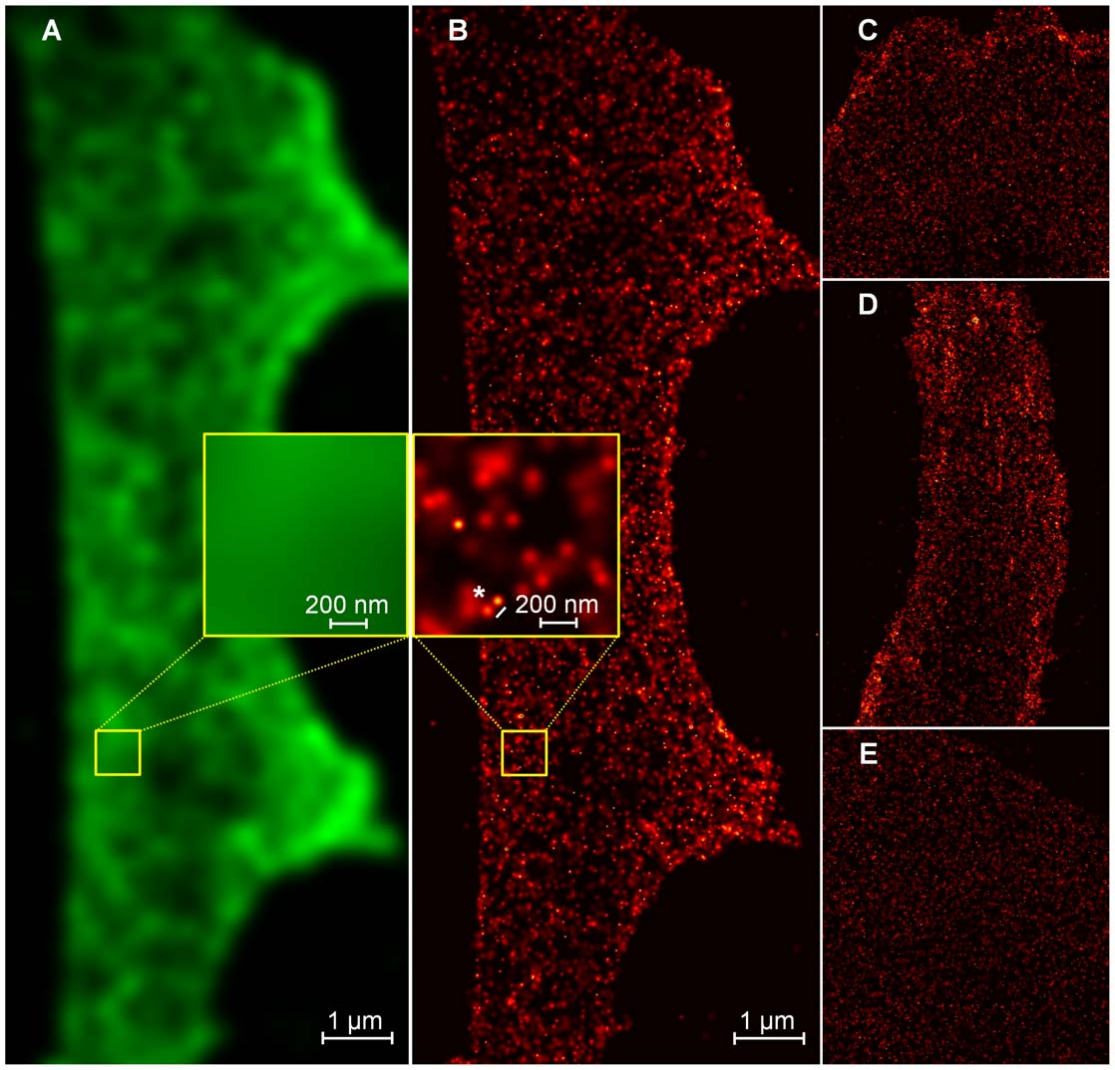
Pgp-GFP fusion protein expression in hCMEC/D3 cells. A: Simulated conventional microscopy image of Pgp-GFP in hCMEC/D3 cells (by convolving a super-resolution image with a Gaussian function with a standard deviation of 200 nm). A conventional microscopy image was not acquired to prevent bleaching. **B:** Super-resolution localization microscopy image of the same cell. Each molecule is convolved with a Gaussian function with a standard deviation equal to its localization accuracy. Some Pgp-GFP molecules could be resoluted with a localization accuracy of approximately 42 nm (asterisk). **C–E:** Three selected images from a total of 20 equivalent images of hCMEC/D3 cells which express Pgp-GFP.

### 2.5 Sample Preparation

Cellular expression of Pgp-GFP (λ_ex_ = 488 nm) in hCMEC/D3 monolayers was monitored after fixation in 4% paraformaldehyde for 10 min, followed by three washes in phosphate buffered saline (PBS) and mounting in ProLong Gold Antifade Reagent (Invitrogen, Karlsruhe, Germany).

**Figure 7:**
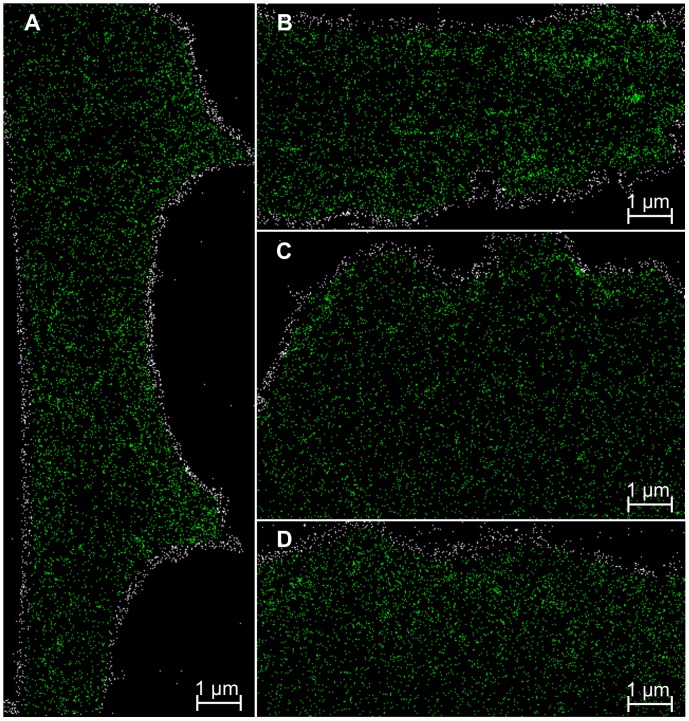
Selected ROIs in the super-resolution localization images. In total, 19,000 Pgp-GFP molecule counts were excluded (white) from the spatial distribution analysis to prevent artifacts due to highly curved cell boundaries.

**Figure 8:**
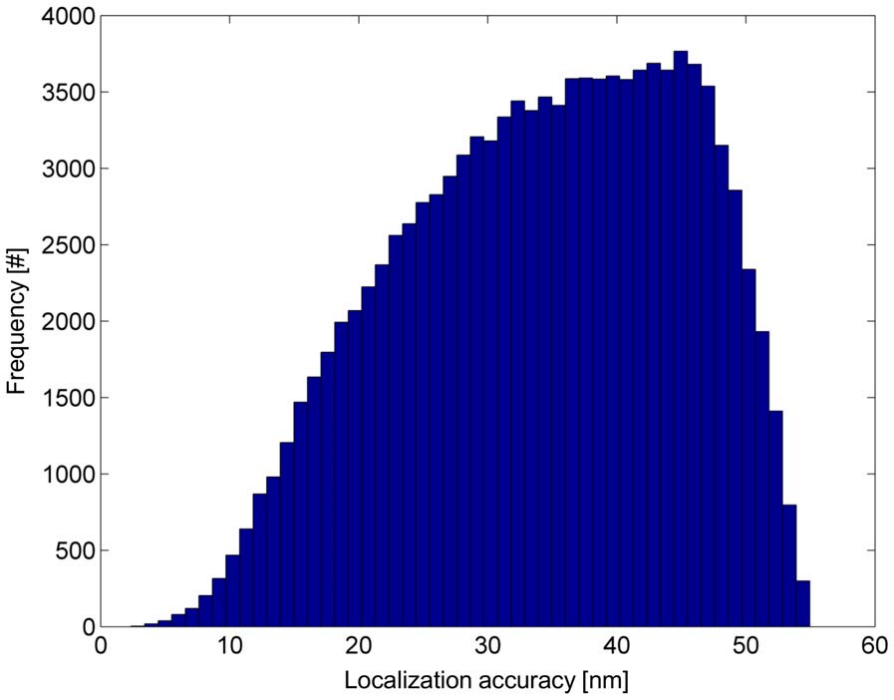
Histogram of the localization accuracy of 112,000 detected single molecules. The average localization accuracy was 34 nm. Note that these 112,000 molecules survived the discarding process and were further kept for spatial distribution analysis.

### 2.6 Spectral Position Determination Microscopy (SPDM)

SPDM is based on the random switching of conventional fluorophores between a very short ‘on’ (fluorescent state) and a long-lived ‘off’ state (reversibly photobleached state) using a single laser source when excited with sufficiently high intensities. This allows separating individual fluorescent molecules in the time domain by acquiring a time series from the same object, each with a sufficiently reduced number of optically isolated fluorescent molecules in the ‘on’ state separated from each other by the conventional resolution limit (200 nm). In each of the individual diffraction patterns obtained, the center is determined by fitting a two-dimensional Gaussian profile to the individual diffraction patterns (i.e. the position of the source fluorescent molecule). Since in typical applications, thousands to millions of positions have to be assigned to obtain a super-resolution image, a computer-based automated reconstruction is required. The localization accuracy is given by the accuracy of the Gaussian fit and thus, is determined by the photon statistic, i.e. the number of detected photons during the integration time [18].

**Figure 9:**
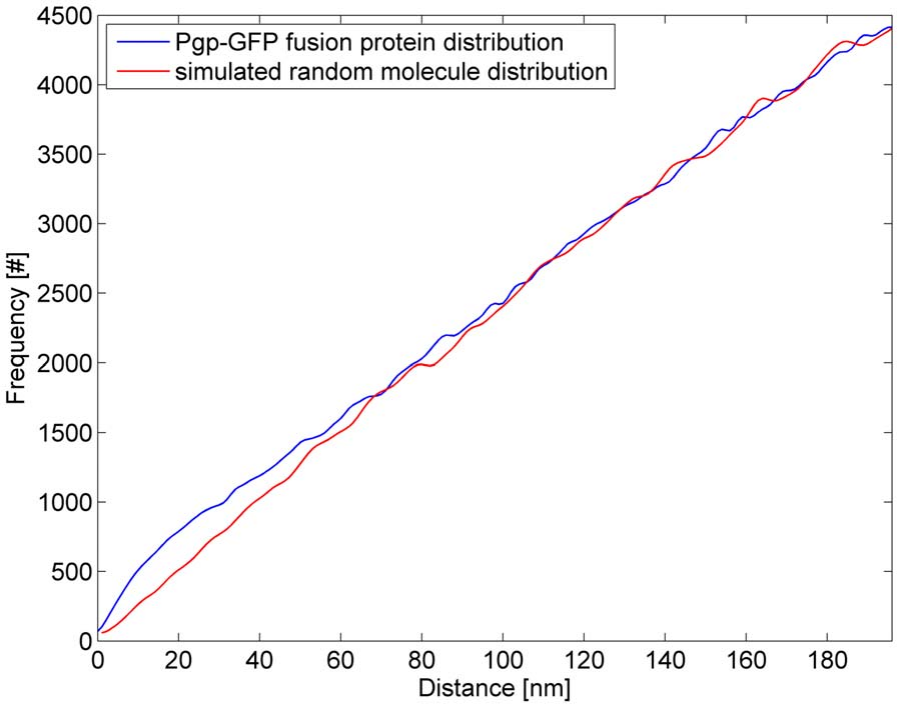
The spatial distribution analysis averaged over the measured cells. Between 5 and 35 nm, a single blurred peak is apparent and converges to a straight line at larger distances. Note the scaling of the y-axis: the peak is caused by several thousands of molecules. This indicates that Pgp-GFP shows clustered formations in hCMEC/D3 cells. The clustered formations themselves are distributed randomly across the cell membrane which can be seen by convergence to the random distribution at larger distances.

The single fluorescent molecule data was generated with the microscope setup described in [19] using a 488 nm laser beam and with only one objective (2D acquisition). The z-resolution of the microscope setup is not sufficient to resolve cell curvatures in the 50 to 150 nm range. Thus, simulations on cell membrane curvatures with respect to a spatial distribution analysis in SPDM images to study this effect are required anyway. Consequently, the z-position was not measured to prevent further photo bleaching. In total, 20 cells have been measured with the SPDM method, each with 2000 frames and an integration time of the camera of 50 ms per frame. Raw data was evaluated by an in-house built software written in Matlab (Matlab, The Mathworks, Natick, Massachusetts, USA). In the first step, the software performs the two-dimensional Gaussian fitting yielding a super-resolution image with single molecule position information. The fitting process was done using an implementation of a Levenburg-Marquardt algorithm [17] by solving the weighted least squares problem between the photon distribution and a model function taking noise into account. It is important to mention that a potential double count of a single fluorescent molecule is prevented. This is done by discarding any other molecule counts within an area of 60 nm and a time frame of 0.5 s (10 images). Secondly, the number of neighboring molecules (frequency) of each molecule at a certain distance is determined (see 2.7 Spatial distribution analysis). In each image a region of interest (ROI) was chosen in a way that the highly curved cell boundary was excluded to from the spatial distribution analysis to prevent interference.

### 2.7 Spatial Distribution Analysis

The newly developed algorithm counts the number (frequency) of neighbor fluorescent molecules of every single fluorescent molecule within a certain distance. The distance increases in steps of 1 nm, from 1 nm to 200 nm, producing a correlation between the distance and the number (frequency) of neighbor molecules at that distance. This is done for every fluorescent molecule in the super-resolution image. Afterwards, the individual correlations of every single fluorescent molecule are summed up and plotted into a histogram for visualization. Histogram data from individual cells are also averaged and plotted in a combined histogram.

### 2.8 Simulations of Molecule Distributions on Curved Cell Membranes

The SPDM setup used here has an improved resolution in the xy-plane, but in the z-direction, the resolution was not improved. Current localization microscopy methods with increased z-resolution are practically still limited and unable to resolve very small curvatures. That means that super-resolution images generated with SPDM are projections over the excitation volume along the z-direction. If these images are then used for analyzing the spatial distribution of the fluorescent plasma membrane molecule Pgp-GFP, the curvature of the cell membrane has a potential influence. Figure 1 visualizes the potential influence of a simulated cell curvature on a projected random molecule distribution. If a curved random molecule distribution is projected along the z-direction, molecule aggregations are induced. These aggregations are not present in the non-curved random distribution. A higher degree of curvature would result in stronger aggregations. To investigate in detail, how different cell curvatures induce aggregations and whether these can be separated from real molecule clustering by spatial distribution analyses, simulations in Matlab (Matlab, The Mathworks, Natick, Massachusetts, USA) were performed.

#### Cell curvature

All simulations were performed using a trimer-cluster with equal inter-cluster-distances of 22 nm. First, the influence of a regular coarse cell curvature was considered. Here, coarse means that the cluster size (22 nm) is small compared to the amplitude (100 nm) and period (160 nm) of the cell curvature. Next, a regular fine cell curvature was simulated to analyze the influence of a curvature period (16 nm) in approximately the same size of the cluster. As both cell curvature types are regular, a curvature with a varying period was chosen to simulate an irregular cell curvature. Transmission electron microscopy images [26] show that the plasma membrane of hCMEC/D3 cells are irregularly curved.

To model the different cell curvatures, two sinusoidal functions with different periods (160 nm and 16 nm) and a chirp function (varying period from approximately 20 nm to 500 nm) were used. The functions are shown in Fig. 2. In summary, the regular coarse curvature is described by a sinusoidal function with a 100 nm amplitude and a period of 160 nm, the regular fine curvature by a sinusoidal function with a 100 nm amplitude and a period of 16 nm and the irregular cell curvature by a chirp function with a 100 nm amplitude and an increasing period from 20 nm to 500 nm. The amplitude for all simulated cell curvatures was chosen between the thickness of the phospholipid bilayer of the plasma membrane (12 nm) and the z-resolution of the SPDM method.

#### Clustering with cell curvature

In this study, seven cases were simulated: clustered and random molecule distribution on a fine cell curvature, clustered and random distribution on an irregular cell curvature, clustered and random distribution on a coarse cell curvature, and finally a random distribution on a non-curved surface. Simulations with random distributions served as controls.

### 2.9 Statistical Analysis of Pgp-GFP Function

The functional experiments were performed at least three times. Calculation of results was accomplished by determining the mean fluorescence intensity (MFI) values from each measurement and calculating the multidrug resistance factor (MAF) for each probe, using the following formula: MAF_Pgp_ = 100 × (MFI_Pgp_ – MFI_0_)/MFI_Pgp_. Data from transduced cells were compared with those from native controls by using unpaired student`s t-test (GraphPad Prism 5.0 software, La Jolla, USA) and expressed as P-value (<0.001***).

## Results

### 3.1 Expression and Function of the Pgp-GFP Fusion Protein as a Pump

The localization of Pgp-GFP fusion protein in hCMEC/D3 cells was determined after viral transduction with the construct. Flow cytometry revealed an expression of the fluorescent protein in approximately 11% of the transduced cells (Fig. 3). To assure that the expressed Pgp-GFP was a functional drug pump, an efflux assay of eFluxx-ID Gold, a newly developed fluorescent substrate for Pgp, was performed in the presence and absence of the selective inhibitor verapamil. The fluorescent probe eFluxx-ID Gold is described to be more sensitive for multidrug resistance (MDR) activity detection than other commonly used probes, i.e. doxorubicin or mitoxantrone [25]. Moreover, eFluxx-ID Gold can be used for multiplex MDR analysis by flow cytometry in GFP expressing cells. To perform that, single stained samples of hCMEC/D3 cells were composed with each dye for electronic compensation correction.

In the absence of verapamil, the transduced Pgp-GFP-hCMEC/D3 cells show remarkably reduced intracellular eFluxx-ID Gold fluorescence intensity compared to nontransduced native hCMEC/D3 cells (Fig. 4A). The intracellular fluorescence intensity of transduced Pgp-GFP expressing cells was 0.06 of the fluorescence intensity of wild type hCMEC/D3, indicating Pgp-GFP-hCMEC/D3 cells had 15.3 times lower eFluxx-ID Gold accumulation. Cells with an additional expression of Pgp-GFP on their membrane accumulated less substrate than those with native Pgp expression, pointing that the expression of the fusion protein was connected with resistance to eFluxx-ID Gold.

In the presence of verapamil, the intracellular fluorescence of eFluxx-ID Gold in Pgp-GFP-hCMEC/D3 cells was considerably increased compared to the hCMEC/D3 cells without verapamil treatment, matching with the expected inhibition of efflux activity of Pgp by verapamil.

Further on, the MDR activity of Pgp-GFP-hCMEC/D3 cells was identified and is increased by a factor of 2 compared to native cells (Fig. 4B).

Summing up, the results approve that Pgp-GFP functions as a drug efflux pump, similar to wild-type Pgp in human brain endothelial cells.

### 3.2 Simulated Influence of Cell Curvatures on Cluster Analysis

Here, the results of the seven cases are presented: clustered and random molecule distribution on a fine cell curvature, clustered and random distribution on an irregular cell curvature, clustered and random distribution on a coarse cell curvature, and a random distribution on a non-curved surface (Fig. 5).

The simulation of clustered and random molecule distributions on a fine sinusoidal cell curvature shows that there is still a peak at 19 nm (Fig. 5), but also a strong periodic modulation in the same order of magnitude resulting in further peaks. This modulation prevents a clear separation between the two phenomena, clustering and cell curvature, since it is not possible to distinguish between the modulation due to the cell curvature and the peak caused by the cluster distribution. In other words, there are also peaks from the cell curvature present in the random molecule distribution. The size of the cluster is in the range of the period of the curvature. Note, that the peak is slightly shifted from 22 nm to 19 nm due to the curvature.

Considering coarse sinusoidal cell curvatures, the situation is different: the random distribution lacks additional peaks and is more or less smooth, because the cell curvature has only a small wavelength. In comparison to the random molecule distribution on a coarse curvature, the peak in the cluster distribution is clearly separable. Coarse curvatures (large period) have no effect when molecule clustering is analyzed. The position of the peak is slightly shifted from 22 nm to 20 nm. Therefore, a quantitative size determination of the cluster without knowledge about the cell curvature becomes difficult.

Finally, the simulation of a chirp function yields an interesting result: similar to a coarse curvature, there are no additional peaks present and the curve is even non-periodic in both the random and the cluster molecule distribution. Additionally, the entire curve is shifted towards smaller distances compared to the random distribution on a non-curved surface. However, the peak is clearly separable since there are no additional peaks in contrast to the case of a fine sinusoidal cell curvature. In the random molecule distribution, no peak is observable and the curve is very smooth. Cluster detection is possible in case of irregular cell curvatures.

### 3.3 SPDM Measurement of Pgp-GFP in hCMEC/D3 Cells

In total, 20 super-resolution images have been generated (according to 1 image per cell). For visualization, selected cell images are shown in Fig. 6. According to the algorithm used, a small percentage of about 2% (3,000 out of 134,000 detected single molecule signals) were considered to be counted twice and consequently were discarded. From the remaining 131,000 single molecules, another 19,000 molecules (14.5%) were discarded by the selection of ROIs to avoid evaluating highly curved cell boundaries (Fig. 7). Over all images, 112,000 single molecules (corresponding to approximately 5,600 Pgp-GFP molecules per cell) have been resolved with an average localization accuracy of 34 nm (corresponding to a resolution of 34×2.35 = 80 nm full width half maximum) (Fig. 8). The spatial distribution analysis averaged over 20 measured cells is shown in Fig. 9. Between 5 and 35 nm, a single blurred peak is visible which converges to a straight line at larger distances. Note the scaling of the y-axis in Fig. 9, the peak is caused by several thousands of Pgp-GFP molecules in clustered formations.

## Discussion

To our knowledge, this is the first report to apply a Pgp-GFP construct on a human brain endothelial cell line to study the spatial distribution on a single molecule level with localization fluorescence microscopy (SPDM). SPDM allows a significantly improved spatial analysis of molecule distributions. Compared to conventional confocal microscopy, single molecule position can be obtained with a resolution far below the diffraction limit of light microscopy while conventional fluorophores and standard fluorescent proteins such as GFP can be used. The position information of hundred thousands of Pgp-GFP molecules, which is provided by SPDM, is essential for a meaningful spatial analysis. In the single super-resolution images (Fig. 6 and 7) molecule clustering or cell curvature effects cannot be seen directly due to the limited detection efficiency of Pgp-GFP molecules by SPDM. However, the detection efficiency was sufficient to find Pgp-GFP molecule clustering (Fig. 9).

It is important to reduce disturbing effects, such as highly curved cell boundaries or potential multi-counting of fluorescent molecules, when the spatial distribution of molecules is investigated [27]. Here, these potential error sources are prevented in two ways before the spatial distribution analysis. First, by discarding every other molecule count within an area of 60 nm and a time frame of 0.5 s (10 images) of another molecule count. Second, by discarding molecules near the cell boundary where high cell curvatures are present due to the attachment of the cells to the chamber slides.

Similar to a recently reported publication [28], the samples were embedded in a standard mounting medium (Prolong Gold) resulting in a longer ‘state to minimize the number of transitions between a long-lived ‘off’ state and the ‘on’ state [29]. Consequently, most Pgp-GFP molecules (98%) are detected only once during image acquisition and no artificial signal clusters are present in the super-resolution image. This is in contrast to a report about an analysis of the distribution of histones with localization microscopy using EGFP in combination with a special ‘switching buffer’ for multiple switching between the ‘off’ and ‘on’ state [30].

The Pgp-GFP fusion protein transduced into hCMEC/D3 cells was shown to be functionally similar to wild type Pgp. Transduction of the functional Pgp-GFP fusion protein in hCMEC/D3 cells enables analysis of expression and localization of Pgp in the BBB. Using a lentiviral vector for transduction of brain endothelial cells results in a high gene transfer efficiency leading to a high protein expression required for spatial distribution analysis.

The simulations of molecule distributions on curved cell membranes show that the spatial distribution analysis to investigate potential clustered formation of molecules in is not influenced by an irregular or coarse sinusoidal curvature of the cell. Only if the cell curvature is fine and periodic (small wavelength), effects of clustering cannot be separated from those of the cell curvature. These small periodic cell membrane curvatures could be excluded in hCMEC/D3 cells, since this would imply that highly ordered cell curvatures are present in these cells which is in contrast to data generated by transmission electron microscopy [26]. Therefore, we conclude that clustered formation of Pgp-GFP molecules in hCMEC/D3 cells is existent. However, no conclusion about the number of constituents forming a cluster can be made since the peak from clustering is blurred due to the limited localization accuracy.

Even if the localization accuracy would be higher and the number of constituents could be resolved, cell membrane curvatures affect the inter-cluster distances preventing a determination of the exact cluster size. Current localization microscopy methods with increased resolution along the z-direction are practically still limited and unable to resolve very small curvatures. With further improvement of the z-resolution, adequate z-position information could be provided to account for cell membrane curvatures during spatial distribution analysis.

The technique will be further developed to analyze the expression pattern of the Pgp model in pathological and disease states, e.g. under inflammatory conditions or diabetes.

In sum, we conclude that Pgp-GFP is distributed in clustered formations in a cell model of human BBB and, based on simulations, the effect of present random cell membrane curvatures can be excluded during spatial distribution analysis. Further, individual formations are distributed randomly over the cell membrane of hCMEC/D3 cells.
